# Normal Treg homeostasis and suppressive function require both FOXP1 and FOXP4

**DOI:** 10.1172/jci.insight.195981

**Published:** 2025-08-12

**Authors:** Dachuan Dong, Vishal J. Sindhava, Ananthakrishnan Ganesan, Martin S. Naradikian, Tom L. Stephen, Andrew Frisch, Kristen M. Valentine, Elizabeth Buza, Karla R. Wiehagen, Michael P. Cancro, Edward E. Morrisey, Haley Tucker, Katrina K. Hoyer, Purvesh Khatri, Jonathan S. Maltzman

**Affiliations:** 1 Geriatric Research Education and Clinical Center, Veterans Administration Palo Alto Health Care System, Palo Alto, California, USA.; 2 Palo Alto Veterans Institute for Research, Palo Alto, California, USA.; 3 Stanford University School of Medicine, Palo Alto, California, USA.; 4 Perelman School of Medicine at the University of Pennsylvania, Philadelphia, Pennsylvania, USA.; 5 Quantitative and Systems Biology graduate program, University of California Merced, Merced, California, USA.; 6 School of Veterinary Medicine, University of Pennsylvania, Philadelphia, Pennsylvania, USA.; 7 Molecular Biosciences and the Institute for Cellular and Molecular Biology, University of Texas at Austin, Austin, Texas, USA.; 8 Department of Molecular and Cell Biology, Health Sciences Research Institute, University of California, Merced, Merced, California, USA.; 9 Institute for Immunity, Transplantation and Infection, Stanford University School of Medicine, Palo Alto, California, USA.

**Keywords:** Autoimmunity, Immunology, Adaptive immunity, Tregs

## Abstract

FOXP3^+^ Treg cells are critical for immune tolerance. Genetic deletion of the Forkhead domain–containing proteins of the FOXP-subfamily member FOXP1 from Tregs results in impaired function associated with reduced CD25 expression and IL-2 signaling, but to date the only other FOXP family member expressed in Tregs, FOXP4, has been minimally studied. To investigate the potential functional interactions among FOXP family members in Tregs, we specifically deleted *Foxp1*, *Foxp4*, or both in FOXP3^+^ committed Tregs in mice. Our findings show that mice with combined, but not individual, deficiency in FOXP1 and FOXP4 exhibit lymphoproliferation, inflammation, autoimmunity, and early lethality. The combined absence of FOXP1 and FOXP4 in Tregs results in an activated/effector-like phenotype with compromised suppressive function in peripheral lymphoid organs, an enhanced germinal center response, and proinflammatory cytokine production. We further show that FOXP1 and FOXP4 bind to *Il2ra* promoter regions to regulate CD25 expression in Tregs. Through pairwise comparison among mouse strains with Treg-specific deletion of *Foxp1*, *Foxp4*, or both, our findings indicate a nonredundant but insufficient role of FOXP4 in Treg function.

## Abstract 2

This study demonstrates that combined Foxp3-specific deletion of both Foxp1 and Foxp4 results in Tregs with decreased IL2Ra expression and reduced *in vivo* function causing systemic inflammation and premature lethality.

## Introduction

Regulatory T (Treg) cells are critical for maintaining immune homeostasis, controlling immune responses, and enforcing self-tolerance. Winged-helix/Forkhead box protein P3 (FOXP3) is a lineage-specific Treg transcription factor (TF) that is indispensable for Treg development and function in both humans and mice (1, 2). Mutations in FOXP3 result in IPEX syndrome in humans and a similar scurfy phenotype in mice (3, 4). FOXP3-associated complexes are required for transcriptional and epigenetic regulation in Tregs but are not sufficient without additional signals (5). Emerging data also suggest that the DNA-binding sites of Forkhead-containing TFs often appear in tandem, providing 2 or more binding sites for FOX proteins (6, 7).

There are 4 members of the FOXP subfamily (FOXP1–FOXP4) with FOXP1, FOXP3, and FOXP4 expressed in lymphocyte lineages (8). Germline deletion of either FOXP1 or FOXP4 is embryonic lethal in mice (9, 10), necessitating a conditional deletion approach for study of these TFs in T cells. FOXP1 maintains a naive phenotype in T cells (11, 12), negatively regulates follicular helper T (Tfh) cell differentiation and germinal center (GC) reactions (13), and is required for maintaining Treg homeostasis and function (14, 15). The role of FOXP4 in T cells has been less extensively studied; T cell–specific loss of FOXP4 leads to decreased cytokine production of CD4^+^ T cell recall responses but has no effect on the development or homeostasis of naive T cells or Tregs (16). Interestingly, proteomic analysis identified FOXP1 and FOXP4 contained within the FOXP3 regulatory complex (17). The presence of both family members in the transcriptional complex and divergent phenotypes with loss of FOXP1 and FOXP4 in non-Tregs suggest a unique function for FOXP4 in Tregs.

To explore the function of these FOXP proteins in Treg cells, we generated mice with Treg-specific deletion of FOXP1, FOXP4, or both FOXP1 and FOXP4. The combined loss of FOXP1 and FOXP4 in committed Treg cells results in increased cellularity of activated T cells, inflammatory cytokine secretion, GC B cell responses, and pathogenic antibody production resulting in systemic inflammation, autoimmunity, lymphoproliferation, and decreased lifespan. Mechanistically, we found that FOXP1 and FOXP4 directly bind to the *Il2ra* promoter and regulate the IL-2/STAT5 axis. These findings highlight specific nonredundant roles for FOXP1 and FOXP4 in Treg homeostatic maintenance.

## Results

### Generation of Treg-specific knockout mice for FOXP1, FOXP4, and FOXP1/FOXP4 double deletion.

To explore the role of FOXP1 and FOXP4 in the regulation and function of FOXP3^+^ Treg cells, we utilized mice with *Foxp1* and/or *Foxp4* alleles flanked by *loxP* sites (*Foxp1^fl/fl^* and *Foxp4^fl/fl^*) (9, 18, 19). To generate Treg-specific knockouts, mice were crossed with a Cre-transgenic strain containing an IRES-YFP-Cre cassette in the *Foxp3* 3′UTR region (*Foxp3^YFP-Cre^*) (20) to generate *Foxp1^fl/fl^* *Foxp3^YFP-Cre^* (referred to hereafter as P1 cKO), *Foxp4^fl/fl^ Foxp3^YFP-Cre^* (referred to hereafter as P4 cKO), and *Foxp1^fl/fl^ Foxp4^fl/fl^ Foxp3^YFP-Cre^* (referred to hereafter as cDKO). To confirm specificity of the knockout, *Foxp3*-positive Treg and *Foxp3*-negative T conventional (Tconv) cells were sorted using the YFP reporter followed by genomic PCR (Supplemental Figure 1A; supplemental material available online with this article; https://doi.org/10.1172/jci.insight.195981DS1) and real-time PCR (RT-PCR) (Supplemental Figure 1B). To control for the presence of the Cre recombinase, we used age-matched *Foxp1*^+*/*+^*Foxp4*^+*/*+^*Foxp3^YFP-Cre^* (referred to hereafter as Cre^Pos^) mice as controls. Genomic PCR and RT-PCR analysis confirmed no evidence of deletion or loss of expression in Tregs compared with Tconv cells from Cre^Pos^ mice (Supplemental Figure 1, A and B).

### Progressive lymphocyte expansion, inflammation, and autoimmune disease in cDKO mice.

**Figure 1:**
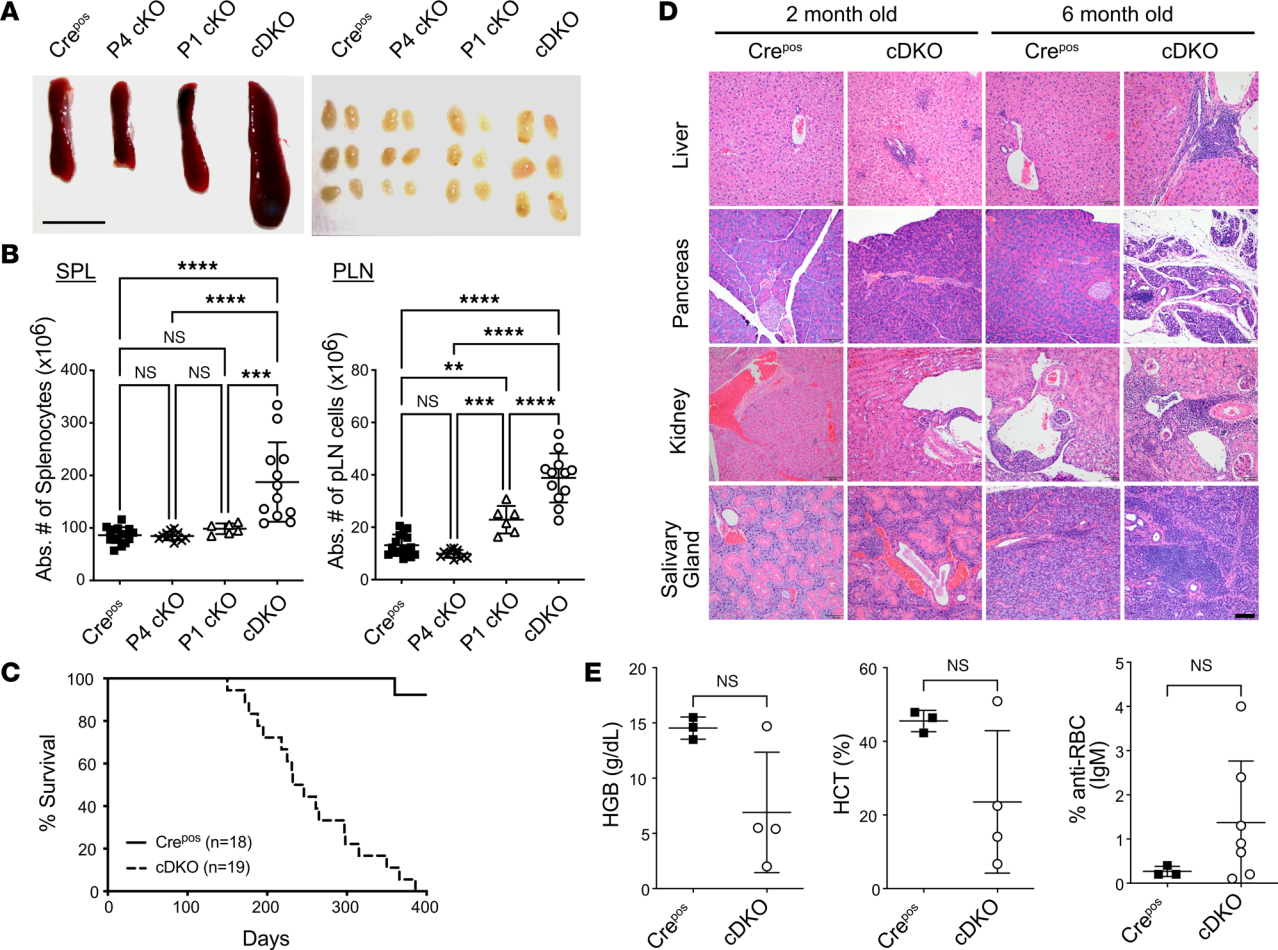
Combined absence of FOXP1 and FOXP4 induces lymphoproliferation and fatal autoimmunity. (**A**) Representative images of spleens (left) and peripheral lymph nodes (pLNs, right) for each mouse strain at the age of 8 to 10 weeks. Scale bar: 1 cm. (**B**) The absolute number of total lymphocytes in spleen or pLN. (**C**) Kaplan-Meier survival curve of the respective mouse strains. (**D**) Representative H&E-stained tissue sections of liver, pancreas, kidney, and salivary gland from 2- and 6-month-old Cre^Pos^ and cDKO mice showing lymphocytic infiltrates. Scale bar: 100 μm. *n* = 2 mice per time point in each group. (**E**) Samples were collected from cDKO mice at a moribund state and Cre^Pos^ at matched time points. Hemoglobin (left) and hematocrit (middle) of the peripheral blood, and anti-erythrocyte IgM antibodies (right) in the serum of cDKO at euthanasia with age-matched Cre^Pos^ littermates. Each symbol represents an individual sample. Data are presented as mean ± SD. Statistical tests: 1-way ANOVA with Tukey’s post hoc test (**B**) and Mann-Whitney *U* test (**E**). NS, not significant. **P < 0.01; ***P < 0.001; ****P < 0.0001.

We first examined the secondary lymphoid organs from 8- to 10-week-old mice of all 4 mouse strains (P1 cKO, P4 cKO, cDKO, and Cre^Pos^). Treg-specific deletion of both FOXP1 and FOXP4 in cDKO mice resulted in splenomegaly and peripheral lymph node (pLN) enlargement (Figure 1A). There was a significant increase in the total number of cDKO splenocytes compared with age-matched Cre^Pos^, P1 cKO, and P4 cKO mice (Figure 1B). Loss of FOXP1 alone was sufficient for an increase in pLN cellularity over that seen in P4 cKO and Cre^Pos^ mice; consistent with the overall size, cDKO mice had more pLN cellularity than P1 cKO, P4 cKO, and Cre^Pos^ (Figure 1B). The increased cellularity in cDKO spleen and pLNs was due to elevated numbers of CD4^+^FOXP3^–^ T, CD8^+^ T, and CD19^+^ B cells (Supplemental Figure 2, A and B). The spontaneous development of lymphoid hypercellularity in cDKO mice was evident as early as 4 weeks of age and became more prominent by 6 months. Together, these data indicate that combined Treg-specific loss of FOXP1 and FOXP4 promotes systemic inflammation.

Together with the altered lymphoid organ cellularity, we noted that some cDKO, but not P1 cKO or P4 cKO, mice were dying prematurely. As such, we performed a longitudinal survival study and found that cDKO mice had a markedly shortened lifespan, with a median survival age of 246 days (Figure 1C). To determine the cause of early lethality, we performed full-body necropsy on 2-month-old and 6-month-old cDKO mice (Supplemental Table 1). Systemic inflammation and multiorgan lymphocyte infiltration occurred in cDKO mice, with higher levels of inflammation observed in 6-month-old cDKO (Figure 1D). By 2 months of age, cDKO mice could be characterized by increased lymphoplasmacytic portal infiltrates found in liver and pancreas of cDKO mice. By 6 months of age, these lesions had progressed from multifocal to coalescing lymphoplasmacytic interstitial infiltrates with extensive acinar-ductal metaplasia. Perivascular lymphoplasmacytic infiltrates were also observed in the renal pelvis and salivary glands of 2- and 6-month-old cDKO mice. These results suggest that Treg-specific deletion of FOXP1 and FOXP4 results in autoinflammatory disease that is milder than that seen when Tregs are entirely absent in FOXP3-mutant scurfy mice and FOXP3^null^ mice (1). Detailed necropsy did not reveal a unifying cause of death. However, at the time of moribundity, a high percentage of cDKO mice displayed a trend toward severe anemia, as measured by hematocrit and hemoglobin levels at the time of death, compared with Cre^Pos^ mice (Figure 1E). cDKO mice also had detectable serum anti–red blood cell (RBC) antibodies, whereas no RBC-specific antibodies were detected in >500-day-old Cre^Pos^ mice (Figure 1E), suggesting FOXP1- and FOXP4-deficient Tregs develop lethal autoimmune hemolytic anemia (AIHA).

### Increased antibody production and GC response in cDKO mice.

**Figure 2:**
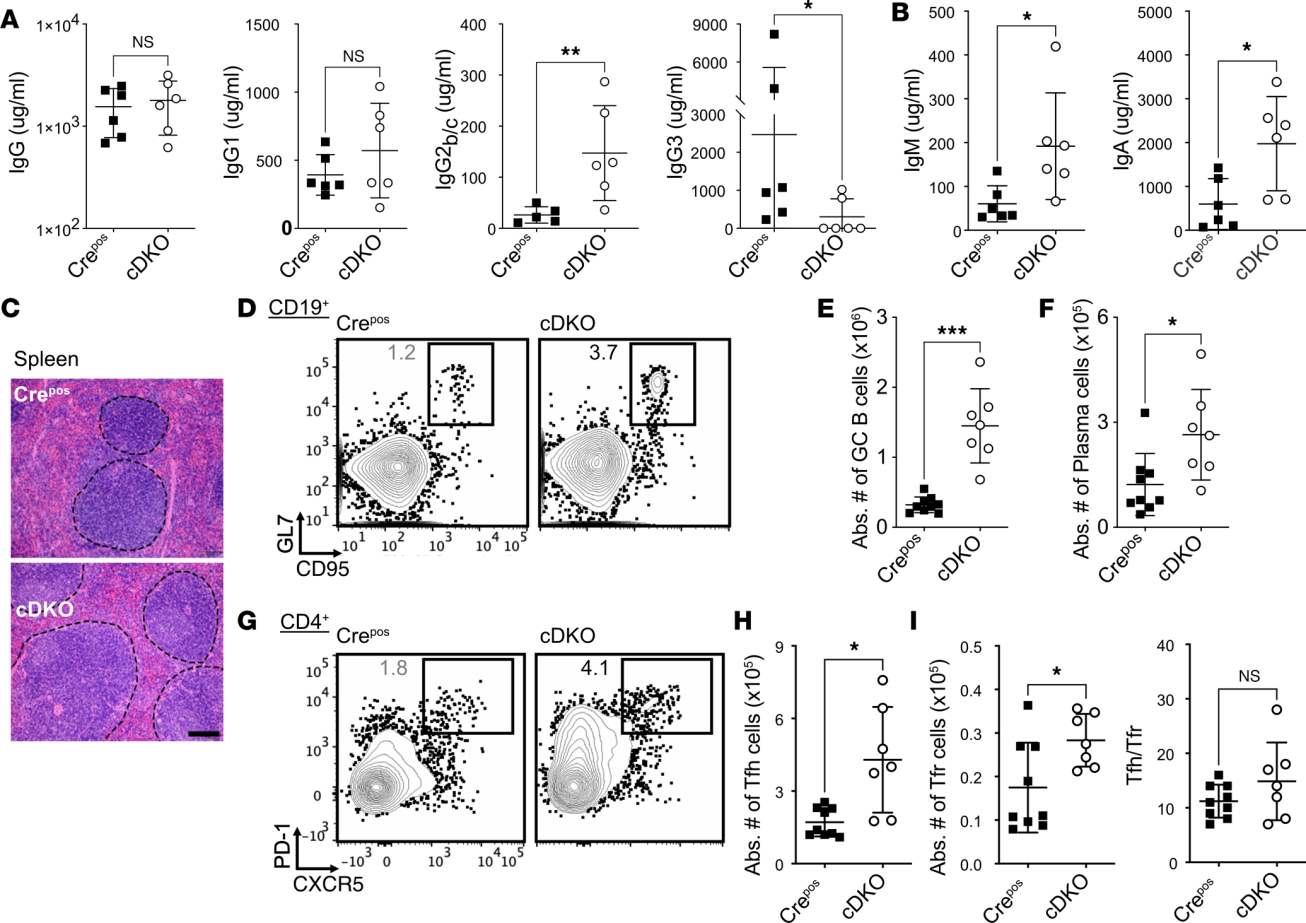
Combined FOXP1 and FOXP4 loss in Tregs results in unrestrained GC response, hyperimmunoglobulinemia, and Tfh cell expansion. Total serum levels of (**A**) IgG, IgG1, IgG2b/c, IgG3, and (**B**) IgM and IgA were assessed by ELISA. (**C**) Representative spleen H&E staining with GCs (outlined with by a dotted lines) from 2-month-old Cre^Pos^ and cDKO mice spleen. Scale bar: 100 μm. (**D**) Representative plots showing flow cytometry analysis of splenic CD19^+^ B cells. CD95^+^GL7^+^ GC B cells are gated. Absolute number of splenic (**E**) GC B and (**F**) CD4^–^CD8^–^GR-1^–^F4/80^–^IgD^lo^CD138^+^ plasma cells in Cre^Pos^ and cDKO mice. (**G**) Representative flow cytometry analysis of CD4^+^TCRβ^+^ gated cells; the gated area indicates CXCR5^+^PD-1^+^ Tfh cells. (**H**) Absolute numbers of Tfh (CD4^+^CXCR5^+^PD-1^+^FOXP3^–^), (**I**) Tfr (CD4^+^CXCR5^+^PD-1^+^FOXP3^+^) (left), and Tfh/Tfr ratio (right) in Cre^Pos^ and cDKO mice. Numbers indicate the percentage of cells in gates (**D** and **G**). Data are representative of 3 independent experiments. Each symbol represents an individual sample. Data are presented as mean ± SD. Unpaired, nonparametric Mann-Whitney *U* test was performed for statistical analysis. NS, not significant. *P < 0.05, **P < 0.01, ***P < 0.001.

Based on the observation of anti-erythrocyte antibodies in the serum of cDKO mice (Figure 1E), we further investigated the regulation of antibody production. We observed a statistically significant increase in IgG2b and IgG2c antibodies, but not total IgG or IgG1 in cDKO mice compared with Cre^Pos^ mice (Figure 2A). IgG3 levels were decreased in the cDKO mice (Figure 2A). Serum concentrations of IgM and IgA were also elevated in cDKO mice (Figure 2B), suggestive of atypical extrafollicular and mucosal plasma cell development. Histological evaluation of cDKO spleen demonstrated larger and more numerous GCs compared with Cre^Pos^ controls (Figure 2C). Consistent with histology, we observed accumulated numbers and frequencies of the CD19^+^GL7^+^CD95^+^ activated GC B cells (Figure 2, D and E) and an increased number of B220^int^CD138^+^ plasma cells (Figure 2F) in the spleen of cDKO mice.

Follicular helper T (Tfh) cells are crucial in the formation of GCs and the establishment of long-lived serological memory (21). The splenic CD4^+^CXCR5^+^PD-1^+^ Tfh cell population was expanded in cDKO mice, in line with the observed GC and plasma cell increases (Figure 2, G and H). One possibility for the increase in Tfh cells is a relative decrease or absence of follicular regulatory T (Tfr, CD4^+^CXCR5^+^PD-1^+^FOXP3^+^) cells (Figure 2I), which originate from thymus-derived FOXP3^+^ cells and act to restrict Tfh cells and GC B cells in vivo (22). Surprisingly, Tfr cells were elevated rather than decreased, with a comparable Tfh-to-Tfr ratio to that in Cre control mice (Figure 2I). Together, these data indicate that Treg deletion of FOXP1 and FOXP4 increases GC responses and Tfh cells under homeostatic conditions and suggests a Tfr cell loss of function rather than absolute loss of Tfr cells.

### Increased turnover rate and absolute number of FOXP3^+^ Treg cells in peripheral lymphoid organs of cDKO mice.

**Figure 3:**
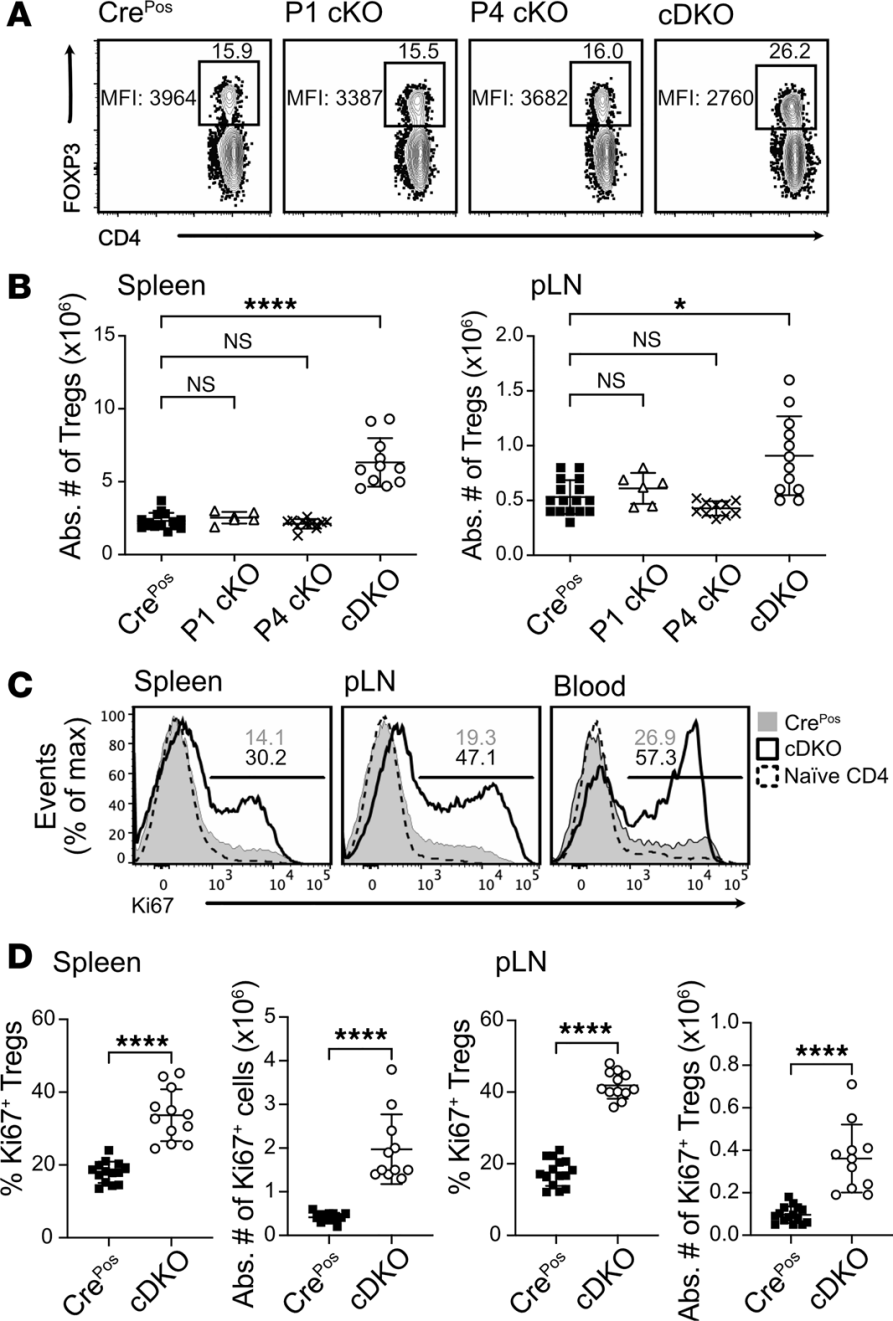
Enhanced turnover and expansion of FOXP3^+^ Tregs in peripheral lymphoid organs of cDKO mice. (**A**) Representative flow cytometry gating strategy used to identify splenic CD4^+^FOXP3^+^ Treg cells from each KO mouse. The percentage of FOXP3^+^ in CD4^+^ T cells and the FOXP3 median fluorescence intensity (MFI) are indicated. (**B**) Total number of FOXP3^+^ Treg cells in spleens (left) and pLNs (right) at the age of 2 months. (**C**) Flow cytometry analysis of Ki-67^+^ cell expression within CD4^+^FOXP3^+^ Treg cells in Cre^Pos^ and cDKO mice. (**D**) Treg frequency and number. Numbers indicate the percentage of cells in gates (**A** and **C**). Data are representative of 3 independent experiments (**A**–**D**). Each symbol represents an individual mouse. Data are presented as mean ± SD. Statistical tests: 1-way ANOVA with Tukey’s post hoc test (**B**) and Mann-Whitney *U* test (**D**). *P < 0.05, ****P < 0.0001.

Lymphoproliferation and inflammation evident in cDKO mice may be attributed to either a relative decreased Treg/Tconv ratio and/or altered function of Tregs. To assess the former hypothesis, we measured the frequency and the absolute number of FOXP3^+^ Treg cells in peripheral secondary lymphoid organs of Cre^Pos^, P1 cKO, P4 cKO, and cDKO mice. cDKO mice had a significantly increased frequency (Figure 3A) and absolute number (Figure 3B) of Tregs in the peripheral lymphoid organs when compared with P1 cKO, P4 cKO, and Cre^Pos^ control mice; cDKO Tregs had a slightly reduced FOXP3 protein level. Conversely, no difference in FOXP3^+^ Treg frequency or absolute number was found when FOXP1 or FOXP4 alone was deleted (Figure 3, A and B).

Treg homeostasis is maintained by T cell receptor–dependent (TCR-dependent) and cytokine-dependent turnover (23–25). To explore the underlying basis of increased Treg cellularity in cDKO mice, we assessed Treg proliferation by measuring expression of the cell cycle–associated proliferation marker Ki-67. The proportion of CD4^+^FOXP3^+^ Tregs positive for Ki-67 was statistically increased in cDKO compared with Cre^Pos^ mice (Figure 3, C and D). These data indicate that the fatal inflammatory disease is not due to a relative or absolute lack of Tregs but rather correlates with an increase in Treg cellularity due to a higher rate of Treg proliferation.

### FOXP1 and FOXP4 are required to maintain normal Treg subsets.

**Figure 4:**
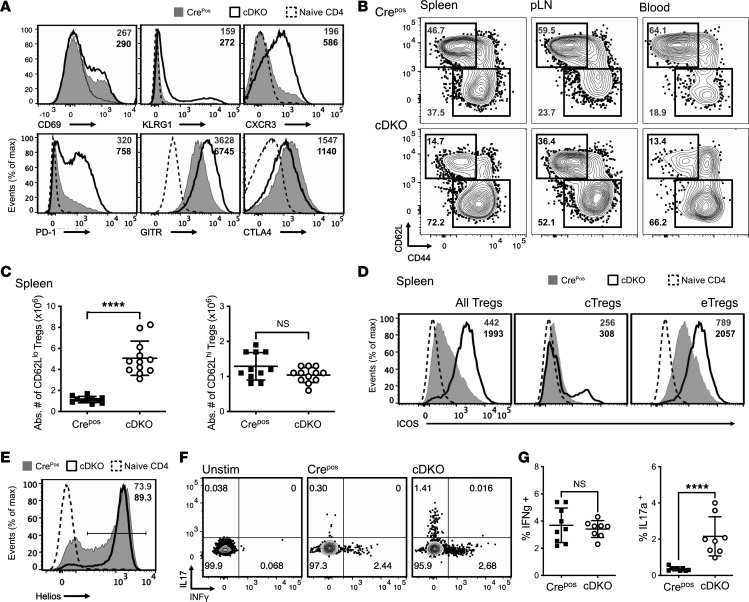
Loss of FOXP1 and FOXP4 changes the phenotype and subsets of Treg cells. (**A**) Flow cytometry analysis of CD69, KLRG1, CXCR3, PD-1, GITR, and CTLA4 surface expression on CD4^+^FOXP3^+^ splenic Treg cells. Median fluorescence intensity (MFI) indicated on plots. (**B**) Representative flow cytometry analysis of cTreg and eTreg subsets in peripheral lymphoid organs and in blood. Gated cell frequency indicated on the plots. (**C**) Absolute number of splenic CD62L^hi^ and CD62L^lo^ Treg cells in Cre^Pos^ and cDKO mice. (**D**) Representative flow cytometry analysis of ICOS expression in YFP^+^ Treg subsets. MFI indicated on plots. (**E**) Flow cytometry analysis of Helios^hi^ subpopulation in CD4^+^FOXP3^+^ Treg cells. Gated proportion indicated on the plot. (**F**) Flow cytometry analysis of frequency and (**G**) quantification of IL-17 and INF-γ production by CD4^+^FOXP3^+^ cells upon PMA/ionomycin stimulation. Data are presented from at least 3 independent experiments. Each symbol in **C** represents an individual mouse. Data are presented as mean ± SD. Significance was assessed with Mann-Whitney *U* test. ****P < 0.0001.

We next explored the direct impact of FOXP1 and FOXP4 deficiency on cell surface phenotypes of Treg cells. Treg cells from cDKO mice showed an activated phenotype, as demonstrated by the elevated levels of CD69, KLRG1, and higher expression of the effector molecules PD-1 and GITR. Furthermore, Treg cells from cDKO mice had increased CXCR3 expression, a chemokine receptor involved in migration to non-lymphoid tissue (26). In contrast, CTLA4 levels in cDKO Tregs were comparable to those in Cre^Pos^ controls (Figure 4A). The enhanced expression of these phenotypic markers was more prominent in cDKO Tregs than Tregs from either P1 cKO or P4 cKO mice (Supplemental Figure 3A). Collectively, these data suggest that the Treg-specific deletion of FOXP1 and FOXP4 increases expression of multiple cell surface activation and effector molecules.

Given that FOXP1 is a transcriptional regulator, and its deletion leads to a moderately activated phenotype in T cells (12, 15), we investigated whether the increased Treg cellularity in cDKO mice was due to the expansion of Treg subpopulations. To this end, we examined the frequency of central memory–like CD44^lo^CD62L^hi^ Treg (cTreg) and effector-like CD44^hi^CD62L^lo^ Treg (eTreg) subsets (27) in various lymphoid compartments. We found a relative expansion in the population of eTregs in the spleen, pLN, and blood of the cDKO mice compared with Cre^Pos^ mice (Figure 4B). P1 and P4 cKO mice also displayed a shift toward eTregs, but to a lesser extent than that seen in the spleens of cDKO (Supplemental Figure 3B). The increased proportion of eTreg subsets in cDKO mice could result from either a decrease in cTregs or an increase in eTregs. We found no statistical difference in the cTreg subset, but rather a substantial increase in eTregs (Figure 4C). Since increased ICOS signals can lead to expansion of the eTreg subset (28), we evaluated ICOS expression and found it elevated in the cDKO Tregs, especially within the CD62L^lo^ compartment (Figure 4D). This suggests that increases in ICOS/ICOS-L signaling may lead to eTreg expansion in cDKO mice. Furthermore, the expansion of the eTreg compartment may alter the composition of the Treg pool in cDKO and contribute to immunodysregulation. A reduction in the Helios^lo^ subset within CD4^+^FOXP3^+^ Tregs indicates a thymus-derived skewed population (Figure 4E). Phenotypically, Tregs in cDKO also exhibited a Th17-like character, further supporting the functional plasticity of the Treg compartment (Figure 4, F and G). Overall, these data demonstrate that FOXP1 and FOXP4 are required to maintain normal Treg quiescence and homeostasis.

### Spontaneous T cell activation and increased production of proinflammatory cytokines in cDKO mice.

**Figure 5:**
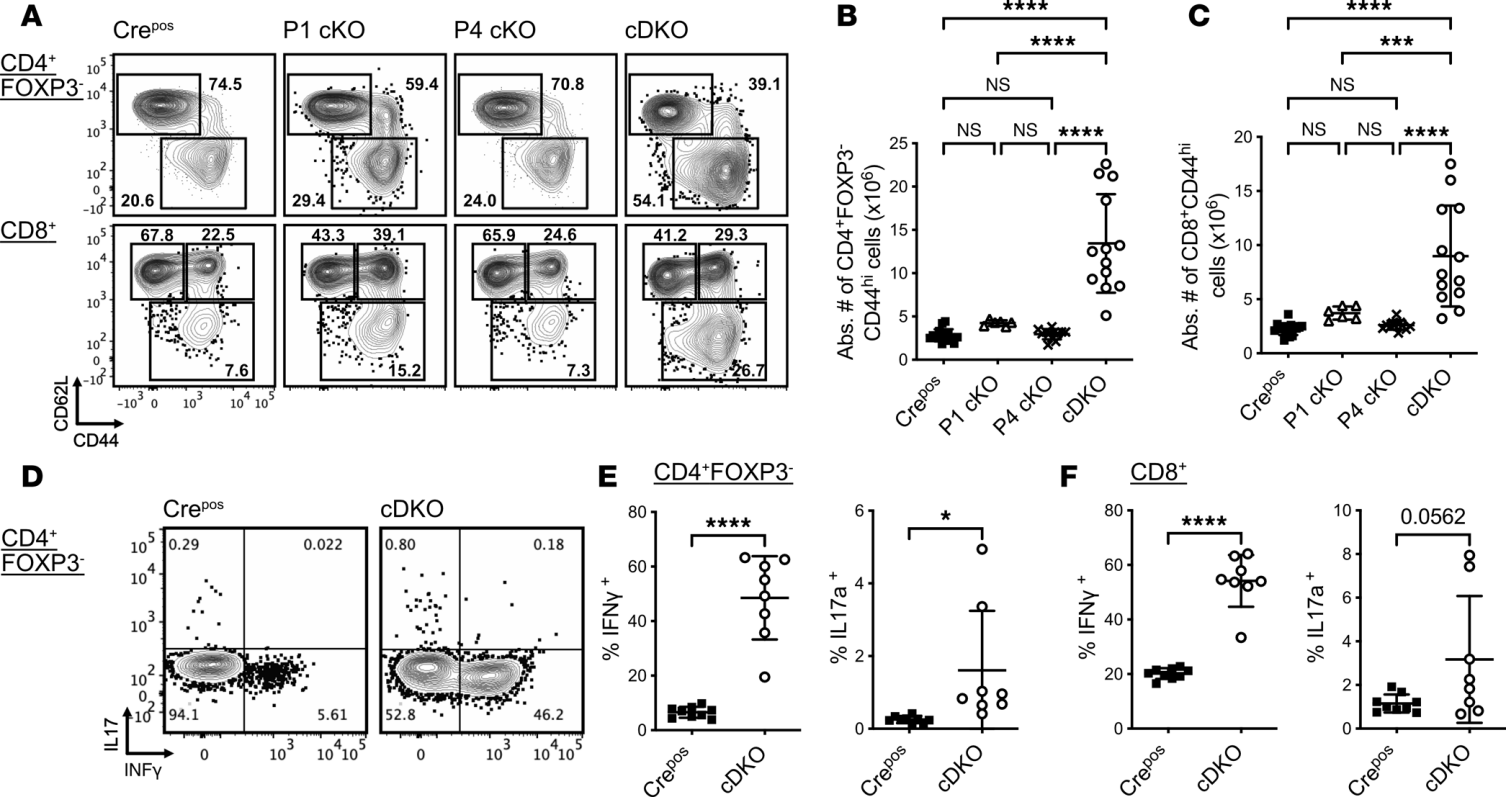
FOXP1 and FOXP4 deficiency cause spontaneous T cell activation and increased production of proinflammatory cytokines. (**A**) Flow cytometry analysis of the frequency of naive and effector/memory subsets in CD4^+^FOXP3^–^ (top) and CD8^+^ cells (bottom) in the spleen from respective mouse strains. (**B**) Number of activated subsets in CD4^+^FOXP3^–^ cells from respective mouse strains. (**C**) Number of activated subsets in CD8^+^ cells from respective mouse strains. (**D**) Flow cytometry analysis of frequency and (**E**) quantification of IL-17 and INF-γ production by CD4^+^FOXP3^–^ cells upon PMA/ionomycin stimulation. (**F**) Quantification of INF-γ and IL-17a production by CD8^+^ cells. Numbers indicate the percentage of cells in gates (**A** and **D**). Data are representative of at least 3 independent experiments. Each symbol represents an individual mouse. Data are presented as mean ± SD. Statistical tests: 1-way ANOVA with Tukey’s post hoc test (**B** and **C**) and Mann-Whitney *U* test (**D** and **E**). *P < 0.05; ***P < 0.001; ****P < 0.0001.

Given the systemic inflammation seen in the cDKO mice, we next evaluated the effect of Treg-specific deletion of FOXP1 and FOXP4 on Tconv (CD4^+^FOXP3^–^) and CD8^+^ subpopulations. Notably, a reproducible increase in the proportion and number of activated/memory T cell population was observed in the splenic CD4^+^ Tconv (Figure 5, A and B) and CD8^+^ compartments (Figure 5, A and C) of cDKO mice compared with Cre^Pos^, P1 cKO, and P4 cKO mice. Both CD4^+^ Tconv and CD8^+^ T cells from cDKO mice also had a significant increase in the Ki-67^+^ proliferating cells compared with Cre^Pos^ mice (Table 1).

In addition, splenic Tconv cells from cDKO mice exhibited a dramatically increased frequency of cells producing proinflammatory interferon γ (IFN-γ) or IL-17 after ex vivo stimulation (Figure 5, D and E), suggesting a propensity to differentiate into Th1- and Th17-like phenotypes that is consistent with class switching of IgG2. Similar observations were seen in CD8^+^ cells (Figure 5F). The increased activation state of T effectors despite increased relative and absolute numbers of Tregs suggests impaired suppressive function of Treg cells in cDKO mice.

### FOXP1 and FOXP4 are required for optimal in vivo suppressive function of Tregs.

**Figure 6:**
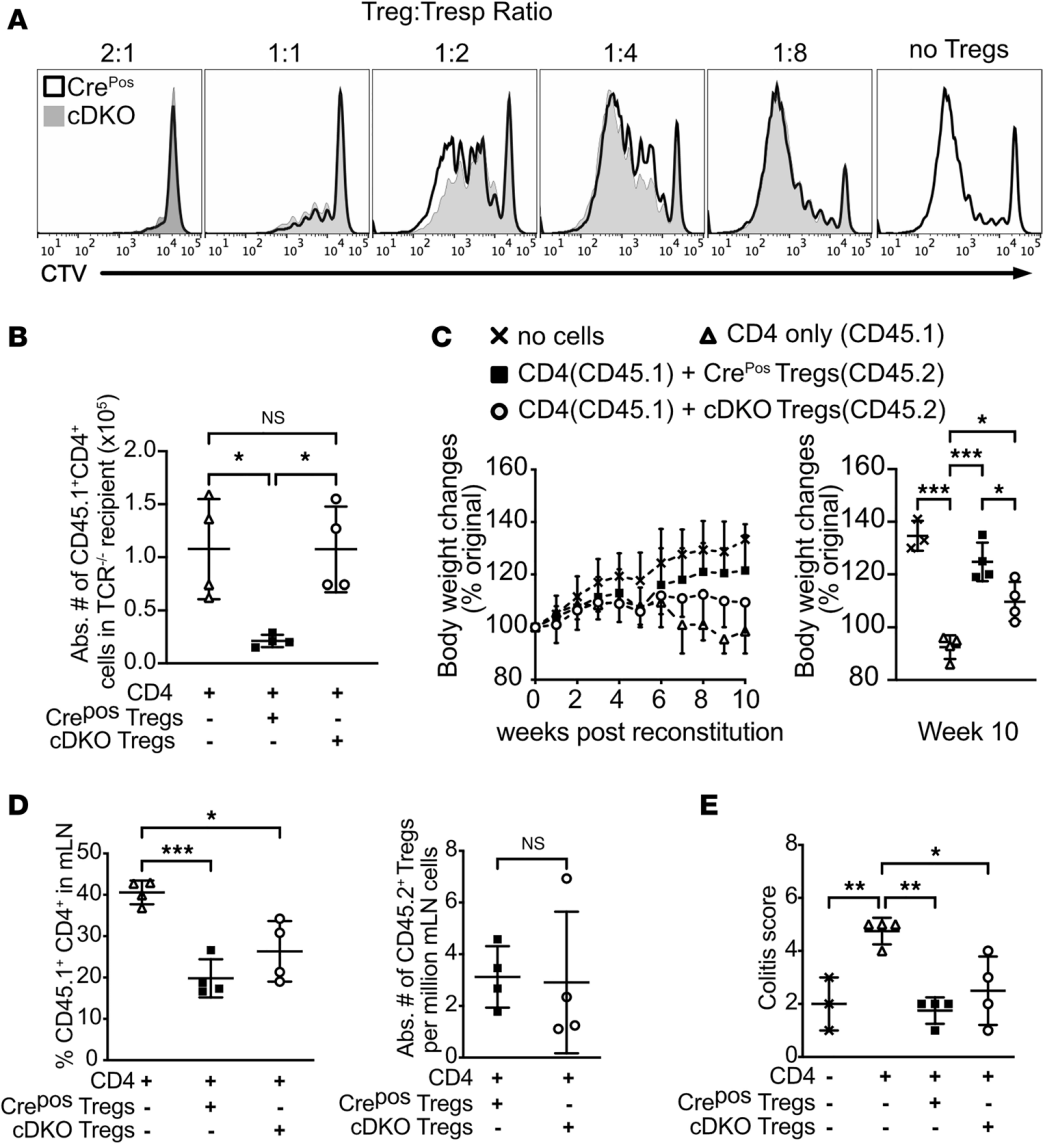
FOXP1 and FOXP4 are essential for Treg suppressive function. (**A**) Representative flow cytometry analysis of CTV-labeled naive T (CD45.1^+^CD4^+^CD25^–^CD44^lo^) cells after 4-day coculture with Tregs (CD45.2^+^CD4^+^YFP^+^) from Cre^Pos^ or cDKO. Representative of 3 independent experiments using a total of 7 cDKO and 7 control mice as the Treg source. (**B**) Absolute number of adoptively transferred CD45.1^+^CD4^+^ cells in the pLNs from TCR^–/–^ recipients on day 7 after transfer is shown. Data are representative of at least 2 independent experiments. (**C**) Changes in body weights of Rag-KO recipient mice after adoptive transfer of CD4^+^ (CD45.1^+^CD4^+^CD25^–^) cells with or without sorted Tregs (CD45.2^+^CD4^+^YFP^+^) from Cre^Pos^ or cDKO. Statistical differences among respective groups were quantified in the 10th week of reconstitution (right), *n* = 5 mice in each group. (**D**) Representative frequency of CD45.1^+^ naive T cells (left) and absolute number of CD45.2^+^ Tregs (right) in mesenteric lymph nodes (mLN) from recipient mice at the end of the study. (**E**) Colitis scores of the respective groups were evaluated in the 10th week of reconstitution. Each symbol represents an individual mouse (except the left panel in **C**). Data are presented as mean ± SD. Statistical tests: 1-way ANOVA with Tukey’s post hoc test (**B**–**E**) and Mann-Whitney *U* test (**D**, right panel). *P < 0.05; **P < 0.01; ***P < 0.001.

As Treg numbers and ratios to Tconv were not decreased, we hypothesized that functional impairment of cDKO Treg cells deficient in both *Foxp1* and *Foxp4* results in the observed inflammatory phenotype. We first assessed the suppressive ability of cDKO Tregs using a standard in vitro suppression assay. Congenic disparate CD45.1^+^CD4^+^CD44^lo^ responder T cells (Tresp) were labeled with cell trace violet (CTV) and cocultured with CD45.2^+^CD4^+^YFP^+^ Tregs sorted from Cre^Pos^ or cDKO mice in the presence of anti-CD3 and irradiated splenocytes. After 4 days of coculture, both Cre^Pos^ and cDKO Tregs suppressed the target CD45.1^+^ Tresp cell proliferation similarly (Figure 6A).

Since in vitro suppression measurements do not always correlate with in vivo function (29), we sought to verify the suppressive function of cDKO Treg cells in an in vivo setting using 2 different Treg dependent models (30): (a) the ability to suppress lymphopenia-induced proliferation (LIP) of T cells (31) and (b) T cell transfer colitis. For LIP, naive CD4^+^ T cells with or without equal number of congenic sorted Tregs from Cre^Pos^ or cDKO mice were adoptively transferred into TCR-deficient mice. Cell numbers in pLNs were assessed on day 7 after transfer. In accordance with previous findings by others (32), the cotransfer of wild-type Cre^Pos^ Tregs prevented naive CD4^+^ T cell expansion. In contrast, cDKO Tregs failed to suppress the expansion of naive CD4^+^ T cells (Figure 6B). To determine cDKO Treg ability to protect from gut-associated inflammation, we adoptively cotransferred congenic CD45.1^+^ naive T cells with sorted CD45.2^+^YFP^+^ Tregs from either Cre^Pos^ or cDKO mice into Rag-KO recipients. There was no statistical difference in body weight change among recipient mice under any condition until 6 weeks after transfer. By the end of the study, a significant weight loss was observed in the groups receiving cDKO Tregs compared with the group receiving Cre^Pos^ control Treg cells (Figure 6C). We also observed a modest increased number of CD45.1^+^ donor–derived T cells infiltrating the mesenteric LNs in the group receiving cDKO Tregs when compared with those receiving control Tregs (Figure 6D). No difference in colitis score was found between cDKO and Ctrl Treg cells at 10 weeks after transfer (Figure 6E). Together, these data indicate a defect in in vivo but not in vitro function of Tregs deficient in both FOXP1 and FOXP4.

### FOXP1 and FOXP4 regulate CD25 expression in Tregs.

**Figure 7:**
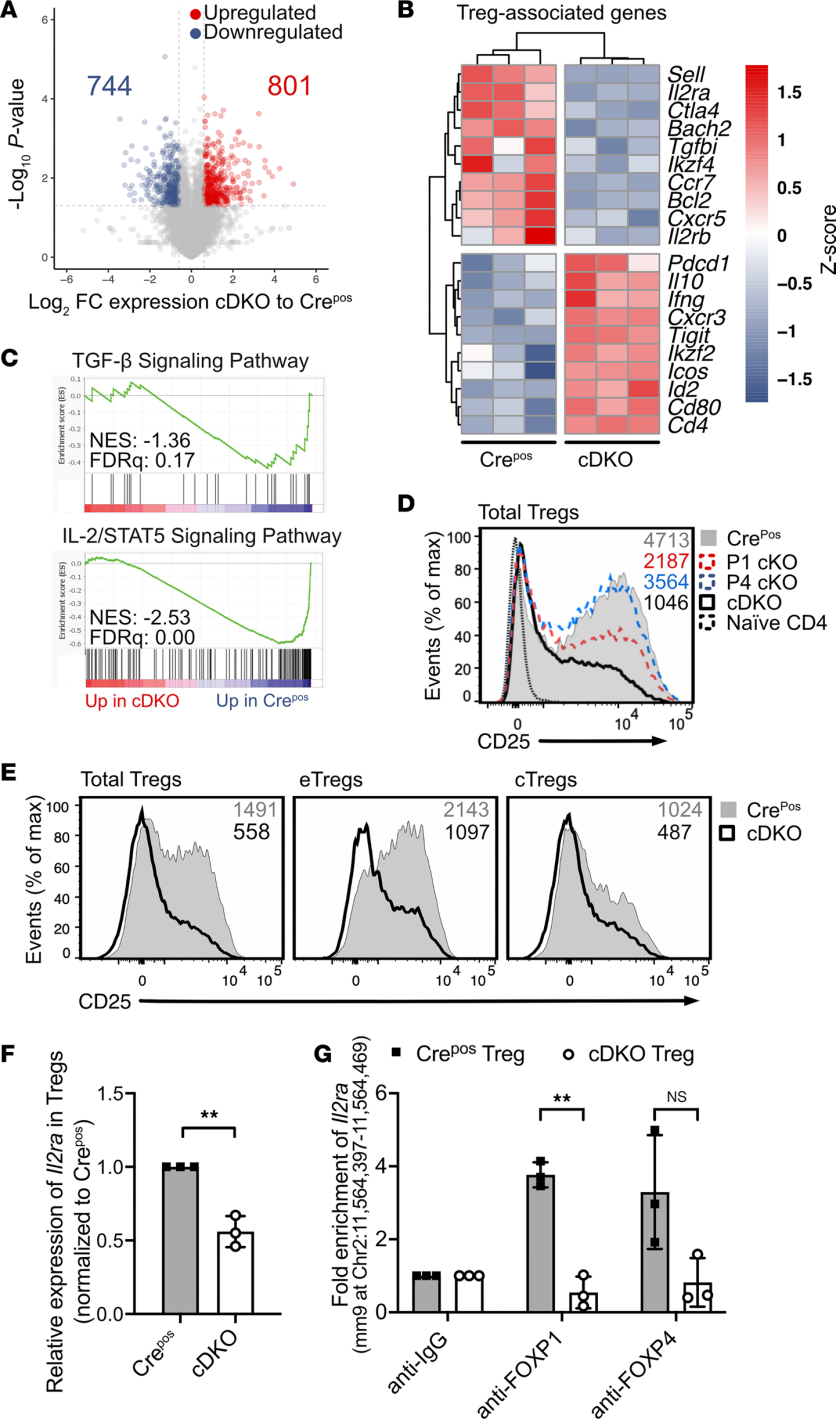
FOXP1 and FOXP4 *trans* regulates *Il2ra* expression in Tregs. (**A**) Volcano plot of the RNA-seq analysis showing 1545 differentially expressed genes in Cre^Pos^ and cDKO YFP^+^ Treg cells. (**B**) Expression heatmap of Treg-associated genes in Cre^Pos^ and cDKO YFP^+^ Treg cells. (**C**) GSEA of TGF-β and IL-2/STAT5 pathways in Treg cells, shown in cDKO/Cre^Pos^. (**D**) Flow cytometry analysis of CD25 expression in CD4^+^FOXP3^+^ Treg cells from the spleen of respective mouse strains. Median fluorescence intensity (MFI) indicated on the plot. (**E**) Flow cytometry analysis of CD25 expression in total Treg, eTreg, and cTreg cells from the spleen of Cre^Pos^ and cDKO mice. MFI indicated on the plots. (**F**) Relative *Il2ra* mRNA expression in sorted Treg cells from Cre^Pos^ and cDKO mice. (**G**) Quantitation of *Il2ra* promoter fragments following immunoprecipitation of DNA from Cre^Pos^ and cDKO Tregs. Data are representative of at least 2 independent experiments and presented as mean ± SD. Each symbol represents an individual sample. Nonparametric Mann-Whitney *U* test was performed for statistical analysis. **P < 0.01.

To elucidate the potential underlying mechanism of the impaired function in cDKO, we profiled the transcriptome of sorted YFP^+^ Treg cells from Cre^Pos^ and cDKO mice. We identified 1545 differentially expressed genes (DEGs, Supplemental Table 2), of which 801 genes were upregulated in cDKO Tregs (Figure 7A). A majority of the upregulated genes, such as *Tigit*, *Icos*, and *Ifng*, were likely in response to activated/effector phenotype and proinflammatory cytokines (Figure 7B). In contrast, genes that contribute to maintaining Treg quiescent status and function were downregulated, including *Sell*, *Il2ra*, *Ctla4*, and *Bach2*. Gene set enrichment analysis (GSEA) found both TGF-β and IL-2/STAT5 signaling pathways significantly attenuated in Tregs from cDKO mice (Figure 7C).

Since the IL-2/IL-2 receptor (IL-2R) axis is crucial for the viability and function of Treg cells (25) and GSEA indicated a decrease in IL-2/STAT5 signaling, we next quantitatively evaluated the expression of the high-affinity IL-2R α chain (IL-2Rα/CD25). CD25 was significantly decreased in FOXP1-deficient Tregs, consistent with a previous report (33), but not in FOXP4-deficient Tregs. Notably, the CD25 relative expression level in cDKO Tregs was even lower than that in P1 cKO (Figure 7D), suggesting that FOXP4 plays a nonredundant role in regulating *Il2ra*. As there was an altered cTreg/eTreg ratio and since CD25 is more highly expressed on CD62L^+^ Tregs than CD62L^–^ Tregs, we further evaluated CD25 expression in the cTreg and eTreg subpopulations and found that both cTreg and eTreg subsets in cDKO have decreased level of CD25 expression (Figure 7E). These data strongly suggest that regulation of CD25 and of the effector phenotype are 2 independent pathways in Treg cells.

To determine whether the decrease in surface CD25 was due to decreased transcription, we analyzed the steady-state level of *Cd25* mRNA by RT-PCR. We observed that Tregs from cDKO mice expressed only approximately half the level of *Il2ra* transcripts as compared with Tregs from Cre^Pos^ mice (Figure 7F), indicating that both FOXP1 and FOXP4 are required for normal levels of *Cd25* mRNA expression. To determine whether FOXP1 and/or FOXP4 directly bind to DNA to regulate transcription from the *Il2ra* locus, we employed chromatin immunoprecipitation followed by PCR (ChIP-PCR) using multiple primer sets (34, 35) to amplify the proximal *Il2ra* promoter. FOXP1 and FOXP4 bound directly to the *Il2ra* promoter region upstream of the transcription start site (–84 to –10). Notably, loss of either FOXP1 or FOXP4 resulted in reduced DNA binding at this region, with the reduction being statistically significant in the absence of FOXP1 (Figure 7G). These data indicate that (a) in the absence of FOXP1 and FOXP4, Treg cells exhibit an effector-like phenotype with attenuated IL-2/STAT5 signaling, (b) FOXP1 and FOXP4 directly bind to the *Il2ra* locus with some degree of functional redundancy, and (c) FOXP4 is necessary but insufficient for the normal function and homeostasis of Treg cells.

## Discussion

In this study, we generated Treg-specific cDKO and compared them with Cre^Pos^ control, P1 cKO, or P4 cKO mice. Phenotypically, mice deficient in both FOXP1 and FOXP4, but neither alone, exhibited an overt lymphoproliferative disease and systemic inflammation. The lymphoproliferation and multiorgan inflammation seen in cDKO mice are widespread but are delayed relative to that observed in FOXP3 mutation or deficiency associated with scurfy mice and IPEX syndromes (3, 36). Tregs in cDKO mice have a high turnover rate and compromised function. Consequently, an unrestrained GC response and proinflammatory cytokine production lead to lethal AIHA. Molecularly, both FOXP1 and FOXP4 can directly bind to the *Il2ra* promotor region regulating CD25 expression in Tregs. Their absence leads to decreased steady-state mRNA and CD25 protein expression. Deletion of FOXP1 alone (P1 cKO) with intact FOXP4 expression cannot maintain normal expression of CD25, potentially explaining the milder phenotype seen in these mice, as published by others (33). Our results indicate a nonredundant but insufficient role of FOXP4 in Treg homeostasis and function.

FOXP1 has been shown to be required for maintaining quiescence in the Treg compartment based on the observation that its deletion in committed FOXP3^+^ Treg cells leads to an increased proportion of activated Treg cells (15), consistent with our findings. Our results also demonstrate that an augmented ratio of activated Treg cells in cDKO mice results primarily from the expansion of CD62L^lo^CD44^hi^ subpopulation, while the CD62L^hi^ populations remain unchanged (Figure 4C). In addition to cDKO mice, several other Treg-specific knockout murine models, including those of IL-2Rα (37), CTLA4 (38), and TGF-β1 (39), share skewing of Tregs toward an activated phenotype mice, indicating the changes are not FOXP specific and supporting the notion that the activated phenotype is more likely the consequence of impaired function of Treg cells combined with cell-extrinsic effects.

Multiple mechanisms have been described through which Treg cells mediate suppression, including CTLA4-mediated trogocytosis and IL-2/IL-2R signaling (40). Despite previous evidence that FOXP1, FOXO1, and FOXO3 can each regulate transcription of CTLA4 (15, 41, 42), we found no decrease in CTLA4 protein levels, suggesting that CTLA4-mediated trogocytosis of CD80/CD86 (38, 43) from antigen-presenting cells is functioning normally. In contrast, loss of function is closely related to the reduction in CD25 expression. Expression of CD25 is crucial for the development, homeostasis, and suppressive function of CD4^+^FOXP3^+^ Treg cells (44). In a model of AIHA induced by adoptive transfer of rat erythrocytes into mice, depletion of CD4^+^CD25^+^ cells prior to AIHA induction increased the incidence of AIHA in C57BL/6 mice. Conversely, adoptive transfer of murine CD4^+^CD25^+^ cells sensitized to rat RBCs prior to induction lessened severity of the disease (45). Furthermore, on a BALB/c background, knockout of either IL-2 (46) or IL-2Rα (47) results in AIHA, with anti-RBC antibodies in the serum. AIHA in the IL-2– and IL-2Rα–KO (and Foxp3-KO) models has been attributed to reduced Treg frequency and function. The absence of FOXP1 and FOXP4 in Tregs leads to an intact compartment but with Tregs expressing lower levels of CD25 on a per-cell basis, suggesting that the AIHA phenotype and subsequent early lethality arise from Treg-intrinsic functional defects related to attenuated IL-2/IL-2R signaling.

The mechanisms by which FOXP proteins coordinate to regulate transcription remain obscure. FOXP-dependent transcriptional regulation occurs through multiple mechanisms, including posttranslational modification of FOXP proteins (48), competitive binding to DNA sites as transcriptional repressors (49, 50), recruitment and assembly of a supramolecular regulatory complex (51, 52), and the formation of spatial domain-swapped dimers (53–55). Assembly of either homo- or heterodimers through 3-dimensional domain swapping (3D-DS) and subsequent effects on transcriptional activity differentiates the FOXP subfamily from other FOX TFs. Domain-swapped homodimers of FOXP1 (56), FOXP2 (57), and FOXP3 (55) have been well characterized by their crystal structures. FOXP1-FOXP4 heterodimers were detected in epithelial cells from mouse skin (58), and transfection approaches have demonstrated formation of human FOXP1-FOXP4 heterodimers regulates the NOTCH signaling pathway in an opposite manner to their homodimeric forms (53), suggesting a potential regulatory role of the heterodimers of FOXP TFs. A lack of direct evidence for FOXP4-FOXP3 dimerization has limited understanding of this potential heterodimeric subtype. But there is still reason to believe that FOXP4 may enable dimerization of and/or with the other 2 FOXP TFs since FOXP proteins are highly conserved in their DNA binding and leucine zipper regions. These 2 important dimerization interfaces strongly infer functional redundancy at the molecular level (59). In Tregs, FOXP1 and FOXP3 share a majority of their binding sites, including *Il2ra*; impaired FOXP3 DNA binding in the absence of FOXP1 was primarily attributed to the inability to form FOXP1-FOXP3 heterodimers (33). In humans, mutation at the DS-dependent leucine zipper hinges in FOXP3 (FOXP3^ΔE251^) compromised its homooligomerization and led to XLAAD/IPEX (52) and can also impair its binding to FOXP1 (60). Similarly, hinge region disruption of human FOXP1 results in its monomeric form (56), suggesting the 3D-DS is essential for FOXP1-FOXP3 heterodimerization, while DS-disrupted FOXP3 (W348Q, M370T, and A372P) has shown a mild impact on *Il2ra* transcription in retrovirally transduced murine T cells with a 72-hour period (55). Our study confirmed that FOXP1 and FOXP4 bind to the *Il2ra* promoter region. Tregs deficient in both FOXP1 and FOXP4, with FOXP3 as the sole FOXP TF, exhibit a significant reduction in CD25 expression compared with P1 cKO mice, further indicating the functional redundancy of FOXP4. Transcription of the *Il2ra* locus is TCR dependent and transient in CD4^+^CD25^+^FOXP3^–^ cells, indicating FOXP3 is not necessary for induction of CD25 expression but may be required for maintenance of expression in FOXP3^+^ Tregs.

**Figure 8:**
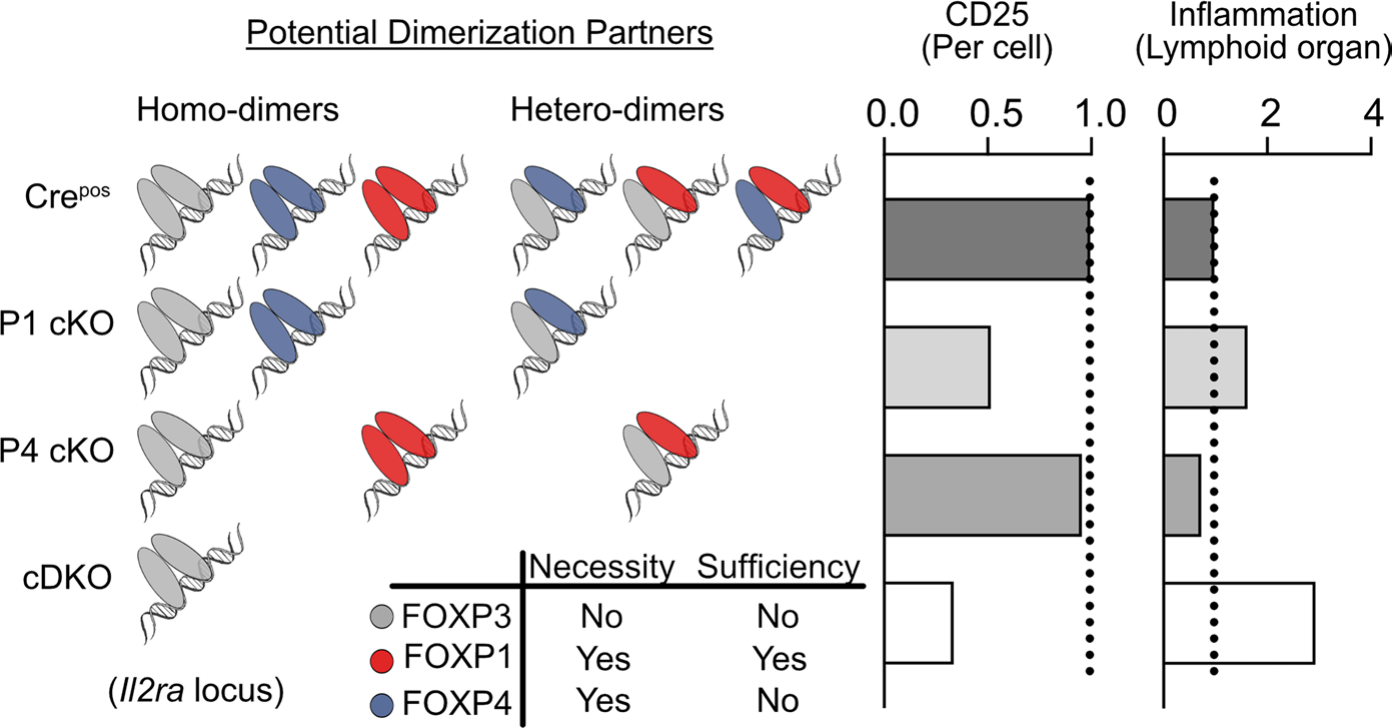
Proposed interaction pattern of FOXP TF members in Treg cells. A schematic view of a potential model of how FOXP1, FOXP3, and FOXP4 interact with each other to regulate gene expression in Treg cells. CD25 was used as an example; the dotted line represents the expression level of CD25 in Cre^Pos^ control mice.

We propose a putative pattern for the dimerization of FOXP TFs and their regulatory capability of the *Il2ra* locus in Tregs (Figure 8). Based on the inference of hydrophobic residues, FOXP3 is more likely to form stable dimers than other family members (61). However, when FOXP3-FOXP3 is the exclusive form of FOXP dimer (as in the case of cDKO), there is a failure to sustain stable CD25 expression. In contrast, in P4 cKO mice, the presence of FOXP1-FOXP1 and/or FOXP1-FOXP3, maintain CD25 expression and thus immune homeostasis. FOXP4-FOXP4 or FOXP4-FOXP3 dimers in P1 cKO mice only exhibit partial functionality regulating the *Il2ra* region, leading to a mild loss of CD25 and mild autoinflammation without overt autoimmune-mediated early mortality.

## Methods

### Sex as a biological variable.

Mice of both sexes were used in this study. Sex was not analyzed as a biological variable.

### Animals.

To generate mice in which FOXP3^+^ cell lineage is selectively deleted for FOXP1, FOXP4, or both, we crossed Cre recombinase knock-in mice line, in which an IRES-YFP-Cre cassette was knocked into the *Foxp3* 3′-UTR region (*Foxp3*^YFP-Cre^) (20), with *Foxp1^fl/fl^* (11) and/or *Foxp4^fl/fl^* (16, 62) to generate P1 cKO, P4 cKO, and cDKO mice. Cre^Pos^ mice were used as control mice for the experiments. Unless otherwise specified, all experiments in the study were conducted using 8- to 10-week-old mice. Mice used in this study were backcrossed with C57BL/6 for 1–2 generations (BC1) and repeated with those backcrossed for 10 generations (BC10); all flow cytometric phenotyping performed on BC1 was similar in BC10 mice. Mice were housed and bred in the University of Pennsylvania and/or the Veterans Affairs Palo Alto Health Care System (VAPAHCS) animal facilities.

### Antibodies and flow cytometry.

The following fluorochrome-conjugated antibodies were used: anti-CD69 (clone H1.2F3), anti–IL-17A (clone TC11-18H10), anti-CXCR5 (clone 2G2), anti-LAG3 (clone C9B7W) (all BD Pharmingen); anti-ICOS (clone 15F9), anti-CD8 (clone 53-6.7), anti-TCRβ (clone H57-597), anti-CD45.2 (clone 104), anti-CD44 (clone IM7), anti-CXCR3 (clone CXCR3-173), anti-PD-1 (clone PM1-30), anti-CCR7 (clone 4B12), anti-Ki-67 (clone 16A8), anti-CD45.1 (clone A20), anti-CD127 (clone SB/199), anti-CD3 (clone 145-2C11), anti-CD62L (clone MEL-14), anti–TNF-α (clone MP6-XT22), anti-CXCR5 (clone L138D7), anti-CD4 (clone H129.19) (all BioLegend); anti-CD25 (clone PC61.5), anti-CTLA4 (clone UC10-4B9), anti-FOXP3 (clone FJK-16s) anti-KLRG1 (clone 2F1), anti-GITR (clone DTA-1), and anti-GL7 (clone GL-7) (all eBiosciences); anti–IL-2 (clone JES6-5H4), anti-CD4 (clone RM4-5), anti–IFN-γ (clone XMG1.2), anti-CD95 (clone J02) anti-CD103 (clone M290), and anti-CD19 (clone 1D3) (all BD Biosciences). For flow cytometric analyses, cells were stained with antibodies against surface antigens at 4°C for 30 minutes in FACS buffer (2% fetal bovine serum [FBS] in PBS). Intracellular staining was performed with the FOXP3 staining kit (eBiosciences) according to the manufacturer’s protocol. Flow cytometry was performed with an LSR II (BD) or sorted on an Aria II (BD), and data were analyzed using FlowJo software (Tree Star).

### In vitro stimulation.

RBC lysed splenocytes from Cre^Pos^ and cDKO mice were stimulated in the presence of brefeldin A with media alone, or with 5 ng/mL PMA and 1 μg/mL ionomycin for 6 hours. At the end of stimulation, cells were harvested and stained for intracellular cytokines using a Cytofix/Cytoperm kit (eBiosciences) according to the manufacturer’s instructions.

### PCR, real-time PCR, and probes.

Sorted splenic naive CD4^+^ (CD4^+^CD44^lo^YFP^–^) T cells or Tregs (CD4^+^YFP^+^) from Cre^Pos^ and cDKO mice were used to extract DNA or RNA. Purified DNA was used to detect wild-type and floxed alleles of *Foxp1*, *Foxp4*, and *Foxp3*^YFP-Cre^ genes by PCR. For *Foxp1*, primers 5′-CTCCTAGTCACCTTCCCCAGT-3′ and 5′-GAACACTGTCGAATGACCCTG-3′ were used to amplify a 280 bp product for the wild-type allele and a 370 bp product for the floxed allele. *Foxp4* genotyping was performed using primer1 (5′-TTTTAAGACCATCTGCGACAAT-3′), primer2 (5′-GATGAGTCAGGGGCTACATAAAAGG-3′), and primer3 (5′-TCAGGAGTGAGGGACCTTATGGT-3′), yielding 420 bp and 556 bp products for the wild-type and floxed alleles, respectively. *Foxp3-Cre* was detected using primers 5′-AGGATGTGAGGGACTACCTCCTGTA-3′ and 5′-TCCTTCACTCTGATTCTGGCAATTT-3′, which amplified a 346 bp product. Total RNA was extracted with RNeasy kit (QIAGEN) and reverse transcribed using SuperScript II Reverse transcription (Invitrogen) according to the manufacturer’s protocols. cDNA was amplified using TaqMan Universal Master Mix (Applied Biosystems) and TaqMan probes for various genes (Applied Biosystems). Real-time PCR was performed with an ABI 7300 (Applied Biosystems). Relative expression was calculated using *Actb* (β-actin) expression as an endogenous control. Fold changes were analyzed with the 2^−ΔΔCt^ method. The following Applied Biosystems Inc. probes were employed: Mm01181991_g1 (*Foxp1*), Mm01269228_g1 (*Foxp4*), and Mm00607939_s1 (*Actb*).

### ELISA.

Ninety-six–well plates were coated with 10 μg/mL anti-Ig (H+L) (Southern Biotech) overnight at 4°C and blocked with PBS containing 2% BSA for 1 hour. Sera were incubated at 1:400 dilutions for 1 hour at room temperature. Detection was conducted using HRP-conjugated goat anti-mouse IgG1, IgG2b/c, IgG3, IgM, or IgA (Southern Biotechnology) diluted at 1:1000 with a TMB substrate kit (BD Biosciences) and color development was quantified using an EMax microplate reader (Molecular Devices).

### RBC antibody assay.

Blood was collected in heparinized and microtainer serum tubes. To assess the presence of anti-RBC antibodies, freshly isolated C57BL/6 RBCs were washed 3 times in PBS and resuspended to a 1% concentration. A 10 μL aliquot of 1% RBCs was incubated with 1 mL of experimental mouse serum at a final dilution of 1:50 for 30 minutes on ice and then washed. Next, RBCs were incubated with anti-mouse IgM-FITC (1:150; (Jackson ImmunoResearch) for 30 minutes on ice. The percentage of antibody-bound RBCs was then quantified by flow cytometry.

### Treg suppression assays.

In vitro: Naive (CD4^+^CD44^lo^CD25^lo^GITR^–^) responder T cells were isolated by FACS from B6.SJL (CD45.1^+^) mice and labeled with Cell Trace Violet (CTV, Invitrogen). CTV labeling was performed by resuspending cells with PBS containing CTV (1 μM) at 37°C by continuous shaking for 5 minutes. The reaction was then immediately quenched with 100% FBS, and the cells were washed before culture. Tregs were sorted from Cre^Pos^ and cDKO mice (CD45.2^+^). CTV-labeled naive CD4^+^ T cells were cultured at various ratios with Tregs in the presence of irradiated, T cell–depleted CD45.1^+^CD45.2^+^ feeder cells and soluble anti-CD3 (1 μg/mL). CTV dilution of naive CD4^+^ (CD4^+^CD45.1^+^) cells was assessed by flow cytometry after 4 days in culture.

In vivo: (a) Lymphopenia-induced proliferation: TCRα-deficient (TCRα KO) mice were adoptively transferred with 1.6 × 10^6^ FACS-isolated naive CD4^+^ (CD4^+^CD44^lo^CD25^lo^GITR^–^) T cells from B6.SJL (CD45.1^+^) mice with or without 0.4 × 10^6^ Tregs from Cre^Pos^ and cDKO mice (CD45.2^+^). Seven days later, pLNs were harvested and the absolute numbers of CD4^+^CD45.1^+^ T cells were determined by flow cytometry. (b) T cell transfer colitis model: 0.5 × 10^6^ FACS-isolated Tconv cells (CD4^+^CD45RB^hi^CD25^–^) from B6.SJL (CD45.1^+^) mice combined with or without 0.5 × 10^6^ FACS-isolated cells from Tregs from Cre^Pos^ and cDKO mice (CD45.2^+^) were transferred into male Rag1-KO (CD45.2^+^) mice. Mice were weighed weekly for the first 4 weeks then twice a week, beginning at 10% weight loss. At the time of euthanizing or 10 weeks after cellular injection, whichever came first, the colitis score was determined based on colon length and histology. In brief, lesions range from 0 to 5 (none to severe): minimal inflammation with slight hyperplasia (score 1) progresses to mild inflammation, possible erosions, and mucin loss (score 2). Moderate inflammation with ulceration (score 3) advances to marked transmural inflammation, ulceration, and mucin depletion (score 4), culminating in severe inflammation with ulceration and gland loss (score 5). Tconv and Tregs percentages were analyzed in the spleen and mesenteric LNs.

### Histology.

Histological analysis of full necropsy specimens was performed in 2- and 6-month-old Cre^Pos^ and cDKO mice by the University of Pennsylvania School of Veterinary Medicine Comparative Anatomy Core facility. Organs from Cre^Pos^ and cDKO mice were isolated, fixed with 10% neutral buffered formalin, and embedded in paraffin. Tissue sections (10 μm) were stained with hematoxylin and eosin (H&E) and examined microscopically in a blinded fashion by a trained veterinary pathologist.

### Bulk RNA-seq and data analysis.

Naturally occurring FOXP3^+^ Treg cells (CD4^+^CD25^+^YFP^+^) were sorted from 2-month-old Cre^Pos^ and cDKO spleens. RNA was isolated using an RNeasy Plus Micro Kit (Qiagen) per manufacturer’s instruction. RNA-seq were carried out on an Illumina HiSeq 2000, generating 100-bp single-end reads. Data were normalized to reads per million. Sequencing data were aligned and annotated to the mouse genome assembly MGSCv37/mm9 at the Stanford Genomic Core. Differentially expressed genes were identified by Cuffdiff, then analyzed, and visualized in R. GSEA was performed as previously described (63). Hallmark gene sets (64) were applied for the analysis.

### ChIP.

FACS-purified YFP^+^ Treg cells from Cre^Pos^ mice were treated with formaldehyde to cross-link protein-protein and protein-DNA complexes. Similarly, isolated cDKO Tregs were used as a genetic negative control. The following precipitation was performed as reported previously (65), with rabbit polyclonal antibodies against FOXP1 and FOXP4 (19). The *Il2ra* promoter fragments present in the immunoprecipitants were quantified with RT-PCR. The sequences of primers used for *Il2ra* promoter quantification were: 5′-TGTGCCTACTCTGTTCTGTGATC-3′ and 5′-GCCAGGAGTTGTTCTATTTAAGCA-3. Results shown for each ChIP condition were analyzed by using the fold enrichment method; the ChIP signals were divided by the irrelevant antibody (Mock IgG) signals, representing the ChIP signal as the fold increases in signal relative to the background.

### Statistics.

Where indicated, *P* values were determined using PRISM software by 1-way ANOVA with Tukey’s post hoc test or 2-tailed nonparametric Mann-Whitney *U* test. *P* values less than 0.05 were considered statistically significant. All graphs show averages of the mean ± SD unless otherwise stated.

### Study approval.

All animal experiments were reviewed and approved by the University of Pennsylvania and/or the VAPAHCS Institutional Animal Care and Use Committees.

### Data availability.

RNA-seq data and normalized gene counts are available at NCBI Gene Expression Omnibus (GEO GSE300286). A complete list of DEGs used in this study has been included in Supplemental Table 2. Values for each figure are included with this manuscript as a supplemental Supporting Data Values file.

## Author contributions

DD and VJS performed experiments, analyzed data, and wrote the manuscript. The order of authorship was based on the relative contributions to this study and manuscript preparation and was determined by mutual agreement. AG and PK performed bioinformatics processing and analysis of RNA-seq data. MSN, TLS, AF, and KMV performed experiments and analyzed data. EB performed necropsies and analyzed histopathological data. MPC conceived experiments and interpreted data. KRW conceived the project. EEM and HT provided reagents. KKH designed anti-RBC antibody experiments, interpreted data, and edited the manuscript. JSM conceived of the project, designed experiments, interpreted data, and edited the manuscript.

## Supplementary Material

Supplemental data

Supplemental table 1

Supplemental table 2

Supporting data values

## Figures and Tables

**Table 1:**
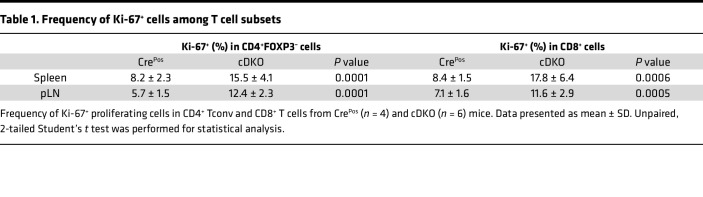
Frequency of Ki-67^+^ cells among T cell subsets

## Version 1 08/12/2025

In-Press Preview

## Version 2 09/23/2025

Electronic publication
