# Supplementary material for: Normal Treg homeostasis and suppressive function require both FOXP1 and FOXP4
Source: JCI Insight. 2025 Aug 12;10(18):e195981. doi: 10.1172/jci.insight.195981 (PMC12487849; doi:10.1172/jci.insight.195981)
Supplement: Supplemental data [file jciinsight-10-195981-s122.pdf]

SUPPLEMENTARY FIGURES

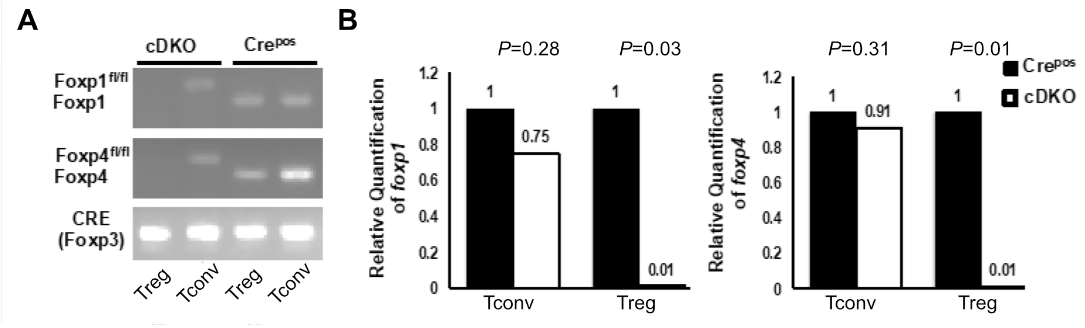

Supplementary Figure 1: Treg-specific deletion of *Foxp1* and *Foxp4* in of cDKO mice. (**A**) DNA-PCR analysis of *Foxp1*, *Foxp4* and *Foxp3*$^{\text{YFP-Cre}}$ in sorted Tconv and Treg cells from Cre^{pos} and cDKO mice spleen. (**B**) Analysis of *Foxp1* and *Foxp4* mRNA expression in sorted CD4^{+} Tconv and Treg cells from the spleen of Cre^{pos} and cDKO mice. Data are representative of three independent experiments (A, B).

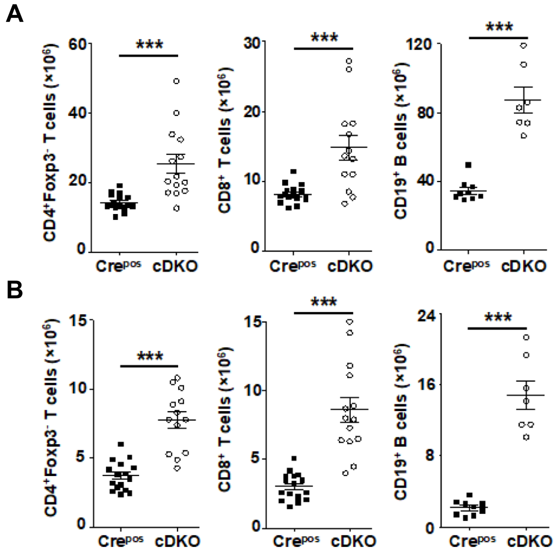

Supplementary Figure 2: cDKO mice develop lymphoproliferation. Total number of $CD4^{+}FOXP3^{-}$ T cells (left panel), $CD8^{+}$ T cells (middle panel) and $CD19^{+}$ B cells (right panel) in the (**A**) spleens and (**B**) pLN of 2 months old Cre^{pos} and cDKO mice. Data are representative of at least three independent experiments. Each symbol represents an individual mouse. ***$P < 0.001$. Data are mean + SEM.

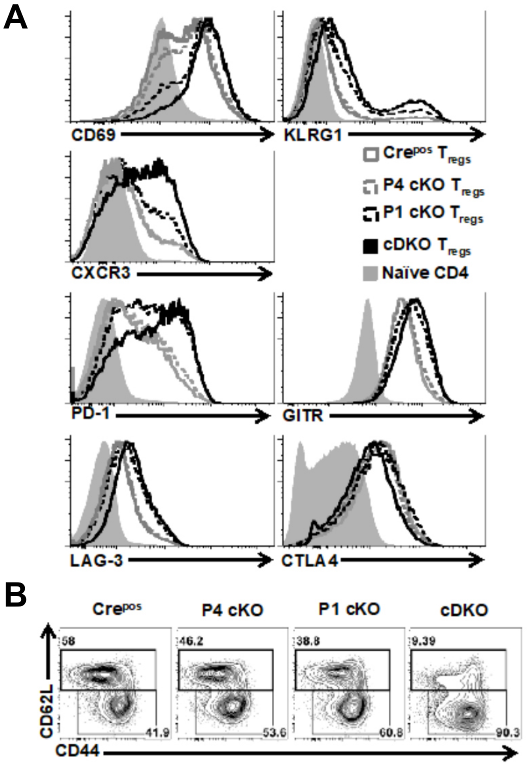

Supplementary Figure 3: FOXP1 and FOXP4 maintain the homeostasis of Treg subset

(**A**) Expression of CD69, KLRG1, CXCR3, PD-1, GITR, LAG-3 and CTLA4 in $CD4^{+}FOXP3^{+}$ Treg cells from the spleen of 2 months old Cre^{pos}, P4 cKO, P1 cKO and cDKO mice. (**B**) $CD4^{+}FOXP3^{+}$ Treg cells from spleen of Cre^{pos}, P4 cKO, P1 cKO and cDKO mice were analyzed for CD62L and CD44 expression by flow cytometry. Gates shows $CD62L^{hi}$ $CD44^{lo}$ cTreg and $CD62L^{lo}$ $CD44^{hi}$ eTreg frequencies of total $CD4^{+}FOXP3^{+}$ Treg cells. Data are representative of at least three independent experiments.
